# Results of Observations on Small-pox in Persons Who Had Been Vaccinated

**Published:** 1845-02

**Authors:** 


*Results of Observations on Small-pox in persons who had been vaccinated.*—These observations were made by Dr. LOSSETTE, in the small-pox ward of the great hospital of Milan. Dr. L. first endeavored to ascertain whether there was any relation between the vaccine cicatrix and its preservative power. With that view, having examined 420 subjects affected with small-pox after vaccination, he classed the cicatrices which they had in three orders, according to their physical characters: 1st, normal; 2d, imperfect; 3d, very imperfect cicatrices. In these 420, 231 had normal cicatrices, 124 incomplete, and 65 only very incomplete. From this it appears that the most regular cicatrices were far from constituting the most certain guarantee against an attack of small-pox.

But does a normal vaccine cicatrix render the consecutive small-pox less confluent? The following table answers this question in the negative.

| Eruption. | Confluent. | Discrete. | Very Discrete. | Total. |
|---|---|---|---|---|
| Cicatrix normal | 83 | 91 | 57 | 231 |
| " incomplete | 53 | 49 | 22 | 134 |
| " very incomplete | 18 | 28 | 19 | 65 |
| | | | | 420 |

Nor do the number of vaccine pustules offer any assurance of protection, as will be seen from the following table:

| Eruption. | Confluent. | Discrete | Very Discrete. | Total. |
|---|---|---|---|---|
| One cicatrix | 30 | 30 | 16 | 76 |
| Two cicatrices | 36 | 35 | 22 | 93 |
| Three cicatrices | 40 | 38 | 20 | 98 |
| Four and more cicatrices | 48 | 65 | 40 | 153 |
| | | | | 420 |

Does the liability to an attack of small-pox, after vaccination, result from the preservative power of the virus having become enfeebled by its successive transmissions? or to the prophylactic powers of this virus, being but temporary and limited to a certain number of years? Dr. L. adopts the latter explanation, and adduces, in its support, the following statistics of 1411 patients, observed in 1837 and 1838, all affected with small-pox.

| | |
|---|---|
| Patients under 5 years of age | 130 |
| " from 5 to 10 | 101 |
| 10 " 15 | 151 |
| 15 " 20 | 203 |
| 20 " 25 | 282 |
| 25 " 30 | 216 |
| 30 " 35 | 160 |
| 35 " 40 | 68 |
| | 1411 |

If we consider that all these patients have been vaccinated in early life, and also the smaller number of individuals who attain the age of thirty years, these statisicts would favor the view of Dr. Losette, of the utility of re-vaccination.—*Amer. Jour.* from *Annali Univ. di Med.*, 1844.

